# Bilateral Sequential Non-arteritic Anterior Ischaemic Optic Neuropathy (NA-AION) Presumed Secondary to 5-Fluorouracil (5-FU): A Case Report and Literature Review

**DOI:** 10.7759/cureus.72754

**Published:** 2024-10-31

**Authors:** Paras Agarwal, Manav Khera

**Affiliations:** 1 Ophthalmology, Warrington and Halton Hospitals NHS Foundation Trust, Warrington, GBR

**Keywords:** 5-fluorouracil (5-fu), bladder tumour, ischaemic optic neuropathy, urologic cancer, retina

## Abstract

Bilateral successive anterior ischaemic optic neuropathy (AION) episodes are uncommon occurrences with a short list of differential diagnoses. We present the case of a 75-year-old male who developed bilateral consecutive non-arteritic AION after commencing 5-fluorouracil (5-FU) chemotherapy. The case report describes a temporal sequence of events with a halt in the progression of signs and symptoms upon cessation of treatment with this agent. Our patient demonstrated preexisting cardiovascular risk factors that were medically well controlled, as evidenced by laboratory results and blood pressure measurements. We reaffirm the current evidence that postulates an association between 5-FU use and arterial vasospasm in blood vessels predisposed to narrowing resulting in prolonged ischaemia to the optic nerve head.

## Introduction

5-Fluorouracil (5-FU) is a chemical agent used to treat a wide range of neoplasms, such as rectal, breast, bladder, and skin cancers [1]. The drug forms a stable complex and prevents the synthesis of deoxythymidine monophosphate (dTMP), resulting in cell cytotoxicity [1]. Numerous adverse effects exist, ranging from diarrhoea and vomiting, resulting in dehydration to side effects such as neutropenic sepsis, which can be fatal even with timely intervention [1]. Mild ocular side effects are uncommon and include excessive lacrimation, conjunctivitis, and blurry vision [2]. Here, we report an unusual side effect of bilateral sequential non-arteritic anterior ischaemic optic neuropathy (NA-AION) in a patient undergoing chemotherapy with 5-FU for bladder cancer.

## Case presentation

A 75-year-old man presented to the eye casualty service with a two-week history of subjective right eye visual blur, mainly in the inferior visual field. There was no associated ocular pain, distortion, or diplopia. The medical history was unremarkable for temporal tenderness, jaw claudication, or polymyalgia rheumatica. The patient’s clinical blood pressure was 130 mmHg systolic and 85 mmHg diastolic, and his recent glycated haemoglobin (HbA1c) level was 46 mmol/mol.

He was recently diagnosed with muscle-invasive T2 stage bladder cancer, for which he had been treated with 5-FU 500 mg/m^2^/day infusion and neomycin one week before the onset of visual symptoms. He also had a history of type 2 diabetes and essential hypertension, for which he was on metformin 500 mg tablet twice daily and ramipril 5 mg tablet once daily, respectively.

At presentation, an ocular examination revealed Snellen visual acuity of 20/40 in the right eye and 20/30 in the left eye. Colour vision assessed with Ishihara plates was also reduced in the symptomatic eye, measuring 2/15 and 13/15 in the unaffected eye. A right relative afferent pupillary defect and a normal, brisk left pupillary reaction were also noted.

**Figure 1:**
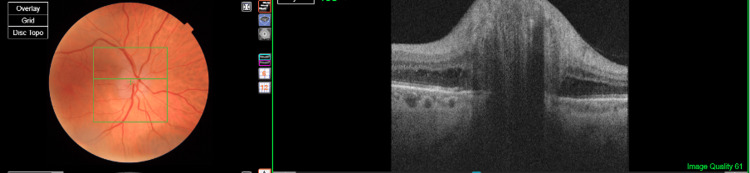
Fundus photo and optical coherence tomography (OCT) scan of the right eye showing poorly defined optic disc margins with evidence of optic disc swelling on OCT.

A dilated fundus examination revealed right optic disc swelling with retinal nerve fibre layer (RNFL) oedema (Figure 1). The retina was flat in both eyes, and the left optic nerve appeared healthy with normal RNFL thickness on optical coherence tomography (OCT) scans (Figure 1). An initial reliable right eye 24-2 Humphrey visual field (HVF) test revealed an inferior reduction in field sensitivity in terms of pattern standard deviation.

Inflammatory blood tests, complete blood count, erythrocyte sedimentation rate, and C-reactive protein were arranged to rule out giant cell arteritis (GCA) as a cause of the suspected NA-AION in the right eye. Blood tests for cardiovascular risk factors, including HbA1c and lipid profile were also requested. Despite having no clinical symptoms of GCA, the patient was also sent for ultrasound Doppler imaging of their temporal arteries and orbital magnetic resonance imaging (MRI) scans of the brain and orbits. The patient’s laboratory investigation results were reviewed three days later. Results are presented in Table 1.

**Table 1:** Laboratory results. ESR: erythrocyte sedimentation rate; CRP: C-reactive protein; HbA1c: glycated haemoglobin; HDL: high-density lipoprotein; LDL: low-density lipoprotein

Blood test	Result	Normal reference range
ESR	10	1–15 mm/hour
Platelet count	237	150–400 × 10^9 ^/L
CRP	<4	0–5
HbA1C	45	20–41 mmol/mol
Total cholesterol	4	<5 mmol/L
HDL-cholesterol	0.85	>1 mmol/L (men) >1.2 mmol/L (women)
Non-HDL-cholesterol	3.85	<4 mmol/L

Temporal artery ultrasound revealed normal arterial flow with no evidence of a halo sign. The orbital and brain MRI images were also unremarkable. The patient’s general practitioner (GP) was advised to optimise his cardiovascular risk factors and a further follow-up was arranged in the neuro-ophthalmology clinic at four weeks.

Two days before his planned ophthalmology follow-up, he returned to the eye casualty service with reports of similar symptoms affecting the left eye. The Snellen visual acuity had decreased in the right eye to 20/50 and the left eye to 20/63. He explained that the second cycle of 5-FU chemotherapy was administered six days before the onset of left eye symptoms. Ocular examination revealed a bilateral reduction in Ishihara colour vision test score of 1/15, with poorly reacting pupils. A dilated fundus examination revealed temporal pallor to the right optic disc; however, the left optic disc showed new RNFL swelling.

**Figure 2:**
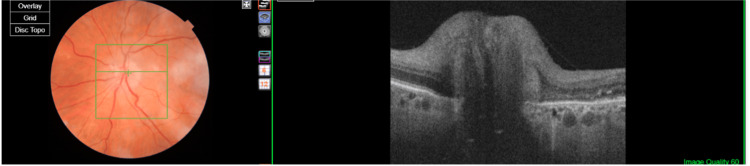
Fundus photo and optical coherence tomography (OCT) scan of the right eye showing poorly defined optic disc margins with evidence of optic disc swelling on OCT.

A 24-2 HVF showed an evolving inferior altitudinal defect, and an OCT scan of the left optic nerve is presented in Figure 2.

Repeated blood tests to assess inflammatory markers and temporal artery ultrasound were requested, all of which returned within normal limits. A discussion regarding the use of oral steroids was initiated with the patient following an explanation regarding the guarded visual prognosis and the likelihood of minimal benefit. However, our patient was keen on a trial, and oral prednisolone was commenced at a dosage of 75 mg once daily in accordance with a 1 mg/kg regimen. Bone protection and a proton pump inhibitor were also prescribed, and a slow wean off of oral steroids was planned.

The patient presented for follow-up two weeks later and his relatives informed us that he did not tolerate the oral steroids. They explained that he became disorientated after the third dose of prednisolone, and after a discussion with the GP, a decision was made to stop the use of oral steroids.

The consideration of bilateral sequential NA-AION as a side effect of 5-FU was considered at this point in the absence of symptoms of GCA, normal inflammatory markers, and two negative temporal artery ultrasound scans. The patient’s oncologist was advised of these ocular findings, and an alternative chemotherapy agent was used without any further ocular side effects.

## Discussion

5-FU is a chemotherapy agent used to treat a wide range of neoplasms, such as rectal, breast, bladder and skin cancers [1,3]. 5-FU is transformed into fluorodeoxyuridine monophosphate, which binds to thymidylate synthase to form a stable complex and prevents the synthesis of dTMP. As dTMP is necessary for DNA replication and repair, its deficiency results in cytotoxicity. The rate-limiting step in the catabolism of 5-FU in both normal and tumour cells is the conversion of 5-FU to dihydrofluorouracil, which is mediated by dihydropyrimidine dehydrogenase (DPD) [1].

The recommended national guideline-approved bladder cancer treatment regimen for 5-FU was 500 mg/m2/day on days one to five, followed by the same dose administered between days 22 and 26 [4]. Our patient’s 5-FU dosage was in line with the above recommendations, and while he had preexisting cardiovascular risk factors, these were medically optimised, as evidenced by blood test results and normal clinical blood pressure. There are in vitro studies suggesting that 5-FU may induce arterial vasospasm in coronary and peripheral arteries, leading to compromised blood flow [5]. In our patient with preexisting vascular risk factors such as essential hypertension, hyperlipidaemia and diabetes mellitus, this may have led to a compounding effect resulting in optic nerve ischaemia. Our case also revealed a temporal relationship between the initiation of 5-FU and the acute development of visual symptoms. In addition, cessation of 5-FU use and switching to an alternative chemotherapy regimen appeared to prevent any further visual deterioration.

A case report by Delval and Klastersky described a similar case, although the patient presented with bilateral optic disc swelling and severe visual acuity reduction at presentation. The patient also had a documented history of DPD deficiency, which is known to induce severe toxicity and adverse events when 5-FU is simultaneously prescribed in this patient cohort [6]. Another patient with optic disc drusen (ODD) developed bilateral optic swelling after receiving leucovorin calcium (folinic acid), fluorouracil, and oxaliplatin (FOLFOX) chemotherapy for bowel cancer. In the absence of cardiovascular risk factors, the combination of compromised blood flow to the vessels supplying the optic nerve head due to ODD and suspected short posterior ciliary artery vasospasm was suggested as a mechanism for bilateral optic disc swelling [7]. Similarly, another patient with unilateral visual reduction, noted to have bilateral optic disc oedema, was observed following the administration of their seventh cycle of FOLFOX chemotherapy for colorectal cancer. In the absence of cardiovascular risk factors, negative carotid duplex ultrasound results and normal cardiac rhythm, the mechanism of bilateral disc swelling was postulated to involve short posterior ciliary artery spasm [8].

## Conclusions

Although infrequent, there are instances of the association between 5-FU use and the development of bilateral optic neuropathy. Our case is unique in this respect, as the patient developed sequential optic disc swelling one month apart, which was directly preceded by the recent administration of 5-FU chemotherapy on both occasions. The incidence of cancer is predicted to rise globally, and chemotherapy regimens that include 5-FU are routinely used in patients with solid malignancies such as bladder, breast, rectal, colon, and pancreatic cancers. This case underscores an uncommon adverse effect associated with 5-FU-based chemotherapy. Clinicians must remain vigilant regarding this possible ocular toxicity, facilitating the timely discontinuation of treatment and prompt referral to an ophthalmologist to prevent visual impairment and harm to ocular tissues.
